# Elevated levels of neutrophil gelatinase-associated lipocalin among OCD patients: an exploratory study

**DOI:** 10.1186/s12888-021-03289-w

**Published:** 2021-05-26

**Authors:** Catarina Raposo-Lima, Inês Miguel Pereira, Fernanda Marques, Pedro Morgado

**Affiliations:** 1 Life and Health Sciences Research Institute (ICVS), School of Medicine, University of Minho, 4710-057 Braga, Portugal; 2 ICVS-3Bs PT Government Associate Laboratory, Braga, Guimarães Portugal; 3 Clinical Academic Center-Braga (2CA), Hospital de Braga, Braga, Portugal

**Keywords:** Obsessive-compulsive disorder, Neutrophil gelatinase-associated lipocalin, Lipocalin, Immunology, Immune dysregulation

## Abstract

**Background:**

Obsessive-compulsive disorder (OCD) is a debilitating psychiatric disease that is characterized by its clinical heterogeneity and complex pathophysiology. This complexity comes from the diversity of pathophysiological factors that have been proposed to be involved in the natural history of the disorder. Many theories on OCD pathology support inflammation as a pathophysiological factor, although studies are not consistent on the presence of a pro-inflammatory state among OCD patients. However, some pre-clinical animal studies suggest lipocalin-2 (LCN2), an analogous form of the acute-phase pro-inflammatory protein neutrophil gelatinase-associated lipocalin (NGAL), may be involved in in the regulation of the stress response, which is thought to be disrupted in OCD.

**Methods:**

Twenty-one OCD patients and 19 healthy subjects participated in this exploratory study. Levels of NGAL were assessed in the peripherous blood of all participants. Severity of disease was assessed using the Yale-Brown Obsessive-Compulsive Scale (Y-BOCS).

**Results:**

OCD patients exhibited significantly higher levels of NGAL when compared to healthy control subjects. No correlation was found between elevated levels of NGAL and severity of symptoms.

**Conclusions:**

This is the first study to report elevated levels of NGAL among OCD patients, adding evidence for a possible role of immune dysregulation in the pathophysiology of OCD.

## Background

Obsessive-compulsive disorder (OCD) is a widely prevalent psychiatric disease that is characterized by the presence of one or two core symptoms, obsessions and compulsions [1]. This chronic and debilitating disease is as heterogenous in presentation as it is in nature, with multiple factors proposed to influence its pathophysiology, from genes to environmental factors, encompassing infection, vitamin deficiencies, stressful life events, brain injury and personality, to cite a few [2]. Since the first report by Swedo et all [3] of a form of OCD arising after streptococcal infection, PANDAS (pediatric autoimmune neuropsychiatric disorder associated with streptococcal infections), many authors have studied the role of inflammation in OCD patients, hypothesizing a role for immune dysregulation in OCD pathophysiology.

In PANDAS, obsessive-compulsive symptoms (OCS) occur in the context of group A ß-hemolytic streptococcal (GABHS) infection, in which anti-streptococcal antibodies are believed to cross-react with epitopes expressed in neuronal cells of the basal ganglia (particularly the caudate and putamen), a key region involved in OCD expression [4–6]. Adding to this hypothesis are studies using GABHS exposure in animal models, which has shown to result in multiple locomotor and cognitive deficits, including anxiety and stereotyped behavior, and increased IgG antibodies and receptors within the striatum, thalamus and frontal cortex, all regions known to be involved in the pathophysiology of OCS (for further details on this subject see the work by Gerentes and colleagues [7] and Mora and colleagues [8]). Although PANDAS might be of relevance for only a minor proportion of OCD cases, this immune/inflammation hypothesis may be significant given the prevalence of OCS among patients with autoimmune diseases [9].

In that regard, many reports on immunology and OCD suggest an increase in proinflammatory markers among OCD patients [7, 10–12], including within the cortico-striatal-thalamo-cortical (CSTC) circuit [13], although recent systematic reviews and meta-analysis point in a different direction, with no differences between OCD patients and controls in what concerns levels of inflammatory cytokines and C-reactive protein [14, 15]. This disparity is due to major methodological differences across studies, with sample sizes, matching of individuals, age of participants, presence of comorbidities and medication status as key limiting factors [10, 14, 15]. Notwithstanding the conflicting literature on the subject, the role of immune dysregulation in OCD should not be overlooked since it might be an important factor in a particular subset of patients, where autoimmunity might be triggered by specific pathogenic agents with similar surface epitopes in the central nervous system (CNS), which may be the case, for instance but not exclusively, of patients with prior history of GABHS infection [16]. Additionally, it is possible that a combination of etiological factors, with specific mechanisms yet incompletely understood, is involved in the development of OCD, and thus the possible involvement of inflammation cannot be ruled out [2].

On this regard, in spite of the inconsistencies across studies and following the hypothesis for the presence of a pro-inflammatory state among OCD patients, we conducted an exploratory study with a small subset of patients by evaluating the levels of plasma neutrophil gelatinase-associated lipocalin (NGAL). NGAL is an acute-phase pro-inflammatory protein [17] that is involved in iron homeostasis [18] and in spine morphology and neuronal excitability in the CNS [19]. Increased levels of NGAL have been detected among multiple sclerosis (MS) patients [20] as well as in heart failure, stroke and coronary artery disease, where it has prognostic significance [21]. It has also been associated with depressive symptom severity [22, 23], although this later finding remains to be proven [24]. In rodents, lipocalin-2 (LCN2) – the analogous form of NGAL – has been shown to be expressed in response to peripheral inflammation [25], focal brain ischemia [26] and spinal cord injury [27, 28]. It is also up-regulated during the active phase of an animal model for MS [20] and seems to be involved in the pathophysiology of Alzheimer’s disease [29, 30] and Parkinson’s disease [31].

Most interestingly, a study using LCN2-null mice showed these animals exhibit anxiety- and depressive-like behavior and cognitive impairments in spatial learning tasks that correlated with increased hypothalamic-pituitary-adrenal (HPA) axis activity and morphological alterations within the hippocampus, specifically, an atrophy of the dorsal hippocampus – which is involved in memory and cognition – and a hypertrophy of the ventral region – which plays a role in emotion. Furthermore, neuronal morphology and dendritic spine density within the hippocampus were altered in mutant mice [32]. This is in accordance with previous studies suggesting a role for LCN2 in the regulation of neuronal morphology and excitability in both the hippocampus and basolateral amygdala in response to stress [33, 34]. Further work with LCN2-null mice demonstrated LCN2 is involved in adult neurogenesis, differentiation and survival, where impairments at this level translate into poor performance in a hippocampal-dependent contextual fear discriminative task [35].

It is noteworthy, however, that both the morphological and behavioral changes observed in LCN-2 null mice are in line with those observed in animals after chronic stress exposure or prolonged administration of exogenous glucocorticoids [36], which raises the question whether or not LCN2 is also involved in the regulation of the stress response, a feature that is known to be disrupted in a number of psychiatric diseases, for instance, OCD.

## Methods

### Subjects

A sample of 40 subjects (21 OCD patients, 19 controls) without previous history of neurological disorders participated in this study. OCD patients were recruited from the Psychiatric outpatient clinic of Hospital de Braga, Portugal, over a period of 3 months, and characterized with a comprehensive clinical assessment. The diagnosis of the disorder was established by experienced psychiatrists, using a semi-structured interview based on Diagnostic and Statistical Manual of Mental Disorders, Fifth Edition (DSM-5). Severity of disease was assessed using the Yale-Brown Obsessive-Compulsive Scale (Y-BOCS) [37, 38]. This clinical assessment allowed for the exclusion of presence of other psychiatric diagnoses, particularly depression and other anxiety disorders. Because one female OCD patient was in clinical remission and asymptomatic at the time of the analysis, this subject was excluded from the study.

All patients were under current pharmacological treatment, primarily a selective serotonin reuptake inhibitor (fluvoxamine, sertraline or fluoxetine).

Healthy controls were recruited to match OCD patients for age and gender.

### Blood sampling and analysis

Serum samples were obtained according to standardized protocols in the Hospital de Braga, Portugal, at the time of recruitment. Human Lipocalin-2/NGAL Quantikine® ELISA Kit (R&D Systems, Inc., Minneapolis, USA) was used according to the manufacturer’s instructions. Briefly, a 96-plate was incubated with 100 μL/per well of Assay Diluent RD1–52, and 50 μL of sample was added to each well. The plate was covered and incubated for 2 h, at 8 °C. Then, it was washed four times with Wash Buffer (400 μL), and 200 μL of Human Lipocalin-2 Conjugate was added to each well. After an incubation step of 2 h (8 °C), the plate was washed four times. At the end, 200 μL of Substrate Solution was added and incubated for 30 min at room temperature, with the plate covered to protect it from light. Finally, after adding 50 μL of the Stop Solution, all samples were quantified by determining optical density in a spectrophotometer set to 450 nm or 450(− 570) nm.

### Data processing and analysis

Statistical analysis for sociodemographic and clinical data was performed using IBM SPSS 27 software [39]. All hypothesis tests had a level of significance (*p*) set at 0.05. Shapiro–Wilk was used to test variables for normal distribution. Since normality was not met, mean comparisons were performed using Mann-Whitney test. Correlations were performed using Spearman’s rank correlation.

### Ethics statement

This study was performed in accordance with the Declaration of Helsinki and was approved by Ethics Subcommittee for the Life and Health Sciences of University of Minho, Portugal, and by the Ethics Committee of the Hospital of Braga, Portugal. All subjects were provided with written informed consent following description of the study goals and procedures.

## Results

### Socio-demographic and clinical characteristics

The groups were similar in respect to age and gender [control group (CONT), *n* = 19; 10 females/9 males; median and interquartile range (IQR) 28.00 (5) years of age; OCD group, *n* = 20; 9 females/11 males; 30.00 (7) years of age] (see Table 1 for further details).

**Table 1:** Sample characterization. Values are presented as mean ± SD for normally distributed variables

		OCD group (***N*** = 20)	CONT group (***N*** = 19)	Statistics
Age^a^		30.50 (7.5)	28.00 (5)	*U* = 178.0; *p* = .746
Sex	Female	45.0%	52.6%	Χ^2^_(1, *N* = 40)_ = 0.227; *p* > .05
	Male	55.0%	47.4%	
Y-BOCS Total		26.10 ± 1.56	–	–
Y-BOCS Obsessions		14.60 ± 0.87	–	–
Y-BOCS Compulsions		11.50 ± 0.76	–	–

*CONT* control, *OCD* Obsessive-Compulsive Disorder, *SD* standard deviation, *Y-BOCS* Yale-Brown Obsessive-Compulsive Scale. ^a^Values are presented as median (IQR) for non-normally distributed variables

Total Y-BOCS score ranged from 9 to 38 (n = 20, mean ± standard deviation (SD) 26.10 ± 1.56, with scores for compulsions and obsession subscales ranging from 4 to 18 and 5 to 20, respectively (compulsions mean score 11.50 ± 0.76 SD; obsessions mean score 14.60 ± 0.87 SD; see Table 1).

### Blood sample analysis

Serum NGAL analysis revealed significantly increased levels among OCD patients relative to healthy participants (OCD, *n* = 20, median (IQR) 91.130 (96.46); CONT, *n* = 19, median (IQR) 60.300 (19.27), with female patients presenting significantly higher levels than female controls (*U* = 18.00; *p < .05*). No differences between male and female participants were apparent within each group (see Table 2 for further information). No correlation was found between NGAL levels and severity of symptoms among OCD patients (total Y-BOCS score r_s_ = .255 (*p* = .278); compulsions r_s_ = .245 (*p* = .299); obsessions r_s_ = .229 (*p* = .332)).

**Table 2:** NGAL levels among participants according to gender. Values are presented as median (IQR) for non-normally distributed variables

Gender	N	OCD group	N	CONT group	Statistics
Male	11	75.430 (50.08)	9	60.92 ± 20.36^a^	*U* = 30.00; *p > .05*
Female	9	142.19 ± 70.39^a^	10	61.75 (31.53)	*U* = 18.00; *p < .05*
Total	21	91.130 (96.46)	19	60.300 (19.27)	*U* = 99.00; *p = .01*

*OCD* Obsessive-Compulsive Disorder, *IQR* interquartile range, *CONT* control. ^a^Values are presented as mean ± SD for normally distributed variables

## Discussion

Until today, the existing work focusing on the role of lipocalin in psychiatric disorders include studies on depression, especially late-life depression, and Alzheimer’s disease. However, and although inflammation has been proposed to be part of the pathophysiological mechanisms behind OCD, with many studies assessing levels of inflammatory markers in OCD patients, no studies to date have included measurements of NGAL levels. In this study, we proposed to assess NGAL levels among OCD patients. So far, this is the first study to do so and to report significant differences between OCD patients and healthy participants, specifically an elevation of the levels of NGAL among patients. We hypothesized NGAL levels would correlate with severity of disease, as it has been observed in studies on depression [23, 40]. However, our data revealed no such association. We have also observed a significant difference in NGAL levels between female patients and controls, but no differences between genders within groups. There are reports of gender differences in inflammatory markers, although not including NGAL, in patients with depressive symptoms and anxiety, which authors hypothesize to be related with different behavioral and inflammatory pathways and possibly with a difference in disease vulnerability [21]. However, our results do not show a difference between genders but a difference among women, and there are no previous reports on gender specific differences in NGAL levels among psychiatric patients. We can only speculate on the variety of factors that might be contributing for our result, for instance differences in disease vulnerability, lifestyle or exposure to environmental factors such as stress. Thus, further studies with larger samples are needed in order to corroborate this result.

Although our findings seem to differ from results from animal studies, where it is the absence of lipocalin, not the elevation, that is behind the abnormal phenotype and neurochemical alterations [32, 35], it is possible that the influence of NGAL follows a U-shaped curve [41], with both the absence and the elevation of levels explaining the behavioral and neurochemical changes. This dual effect has been proposed regarding inflammatory cytokines and their impact on sleep, but this model is yet to be proven [42]. However, studies assessing NGAL function have provided interesting results supporting this notion, where both lipocalin deficiency and overexpression are associated with behavioral impairments. Thus, while a number of animal and human studies have found elevated levels of lipocalin in disease states such as depression [22, 40], schizophrenia [43], multiple sclerosis [20], Parkinson’s [31] and Alzheimer’s disease [29, 30], suggesting lipocalin overexpression might play an important role in mediating the observed deficits, other authors have reported findings such as increased anxiety- and depressive-like behavior and cognitive impairments displayed by LCN2-null mice [32, 33, 35, 44]. On the contrary, a recent study by Mike and colleagues assessing the role of LCN2 on the neurobehavioral manifestations of a mouse lupus model found LCN2 deficiency in Sle1,3 mice attenuated the depressive-like phenotype as well as memory impairments that are characteristic of the model. Most notably, LCN2-KO animals did not exhibit impairments in exploratory behavior or motor coordination nor showed deficits in the sucrose-preference, object recognition and object placement tests compared to control animals [45], which is in line with other reports where both LCN2 deficient and wild type mice exhibited a similar behavioral phenotype in response to liposaccharide injection [46]. Thus, it seems plausible to admit lipocalin may play different roles according to the physiology or pathological conditions [47, 48] and further studies are needed to better understand its role in mediating disease states.

Most importantly, since lipocalin constitutes a marker of inflammation not necessarily associated with infection [48], our results highlight the importance of describing the inflammatory profile of OCD patients, given this might be of relevance for the understanding of the disorder in patients other than those evidencing OCS in the context of prior history of infection. Previous studies have demonstrated how other immune abnormalities might be related to glutamatergic dysfunction within the CSTC circuit, the main pathway associated with OCD development [16, 49]. Additionally, animal and human studies have shown lipocalin to be expressed in a variety of brain regions, including the striatum, thalamus and prefrontal cortex [31, 50], regions that have been implicated in OCD pathophysiology. The link between immune dysregulation and OCD is yet to be unraveled, although theories suggest glutamate might play a strategic role given its presence within the CSTC circuit and its involvement in regulating T cell function [51, 52]. Given the hypothetical nature of such theory, further work is needed in order to better understand the relative contribution of immune dysregulation in OCD pathophysiology, with larger samples, detailed clinical characterization of participants, control for medication effect and rigorous selection criteria as key points to have into account in future studies.

Naturally, our study has some limitations. The use of a small number of patients limits the generalization of results. Additionally, whether the observed increase in NGAL levels is a cause or a consequence of the disorder remains to be defined and should warrant further investigation. Future studies on this subject would also benefit of a more detailed assessment of the inflammation status of subjects, with inclusion of other markers of inflammation and the determination of antistreptolysin titers. Thus, it would be interesting to proceed with studies on this subject not only by including larger, well characterized cohorts, but also through the use of animal models, which allow for the dissection of biological mechanisms otherwise impossible to study in humans.

In conclusion, we herein report differences in NGAL levels among OCD patients compared to healthy controls. We offer additional evidence to the immune dysregulation hypothesis of OCD by reporting elevated levels of NGAL among OCD patients versus healthy controls, with higher differences being found among women.

However, while our results warrant further replication, more research is needed in order to clarify the role of NGAL in this neuropsychiatric disorder.

## Data Availability

The datasets used and/or analyzed during the current study are available from the corresponding author on reasonable request.

## Abbreviations

CNS: Central nervous system
CSTC circuit: Cortico-striatal-thalamo-cortical circuit
HPA axis: Hypothalamic-pituitary-adrenal axis
LCN2: Lipocalin-2
MS: Multiple sclerosis
NGAL: Neutrophil gelatinase-associated lipocalin
OCD: Obsessive-compulsive disorder
OCS: Obsessive-compulsive symptoms
Y-BOCS: Yale-Brown Obsessive-Compulsive Scale
